# (*E*)-6,6′-(Diazene-1,2-di­yl)bis­(1,10-phenanthrolin-5-ol) tri­chloro­methane disolvate: a superconjugated ligand

**DOI:** 10.1107/S205698901900954X

**Published:** 2019-07-23

**Authors:** Muhib Ahmed, Michael Devereux, Vickie McKee, Malachy McCann, A. Denise Rooney

**Affiliations:** a Department of Chemistry, Maynooth University, Co. Kildare, Ireland; b The Centre for Biomimetic & Therapeutic Research, Focas Research Institute, Technological University Dublin, City Campus, Camden Row, Dublin 8, Ireland; c Department of Physics, Chemistry and Pharmacy, University of Southern Denmark, Canpusvej 55, 5230 Odense M, Denmark; d School of Chemical Sciences, Dublin City University, Glasnevin, Dublin 9, Ireland

**Keywords:** crystal structure, diazo, phenanthroline, superconjugated ligand

## Abstract

Phenanthroline ligands are important metal-binding mol­ecules which have been extensively researched for applications in both material science and medicinal chemistry. Azo­benzene and its derivatives have received significant attention because of their ability to be reversibly switched between the *E* and *Z* forms and so could have applications in optical memory and logic devices or as mol­ecular machines. Herein we report the formation and crystal structure of a highly unusual novel diazo-diphenanthroline compound, C_24_H_14_N_6_O_2_·2CHCl_3_.

## Abstract 2

The preparation and structural characterization of the diazo-diphenanthroline compound, (*E*)-6,6′-(diazene-1,2-di­yl)bis­(1,10-phenanthrolin-5-ol) are described. The fully conjugated bis-phenanthroline mol­ecule is expected to offer exciting new physical and chemical properties, and should form the basis of novel metal coordination complexes as a consequence of the dual *N*,*N*′-1,10-phenanthroline chelating moieties situated on the opposite ends of the mol­ecule.

## Chemical context   

The chemical versatility of 1,10-phenanthroline (phen), its substituted derivatives and corresponding metal complexes (Bencini & Lippolis, 2010 ▸) is exemplified by their uses as organic light-emitting diodes (OLED)/electroluminescent display and solid-state lighting materials (Li *et al.*, 2009 ▸), fluorescence mol­ecular probes and imaging agents (Haraga *et al.*, 2018 ▸), ion sensors (Zheng *et al.*, 2012 ▸), solar energy converters (Freitag *et al.*, 2016 ▸), anti-cancer and anti­microbial cytotoxins (McCann *et al.*, 2012 ▸), DNA/RNA binding/cleavage (Kellett *et al.*, 2011 ▸), enzyme inhibitors (Zhu *et al.*, 2015 ▸), biomimetics (Casey *et al.*, 1994 ▸) and catalysts (Lu *et al.*, 2015 ▸). Given the resourcefulness of phenanthrolines, there is a continuing demand for new mol­ecules containing this structural motif.
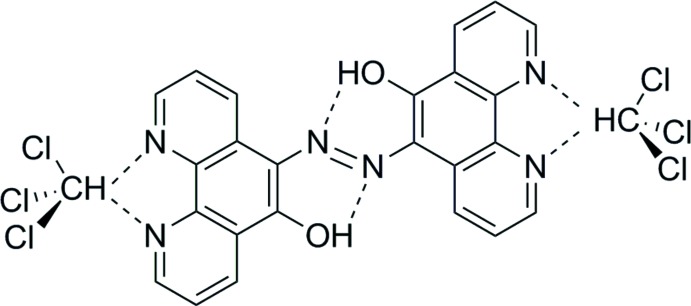


Herein, we detail the preparation and structural characterization of the purple diazo-diphenanthroline compound, (*E*)-6,6′-(diazene-1,2-di­yl)bis­(1,10-phenanthrolin-5-ol) (**1**), which was isolated in low yield from the reaction between 1,10-phenanthroline-5,6-dione (phendione) and isonicotinic acid hydrazide (isoniazid). Compound **1** crystallizes with two tri­chloro­methane solvate mol­ecules (**1**·2CHCl_3_). The fully conjugated bis-phenanthroline mol­ecule **1** is expected to offer exciting new physical and chemical properties as a stand-alone organic mol­ecule, and it will also birth a plethora of inter­esting metal coordination complexes as a consequence of the dual *N,N′*-1,10-phenanthroline chelating moieties situated on the opposite ends of the mol­ecule.

## Structural commentary   

**Figure 1:**
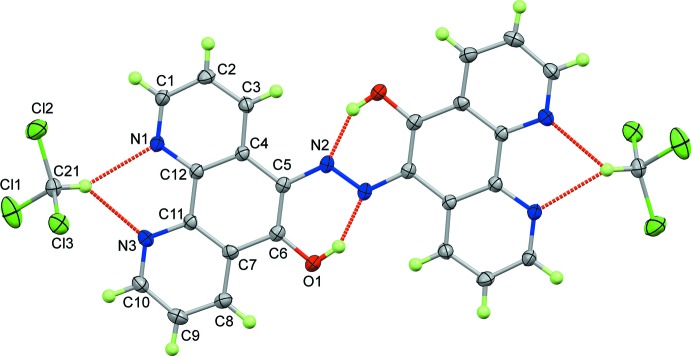
Perspective view of **1**·2CHCl_3_ showing the labelling scheme for the asymmetric unit with displacement ellipsoids drawn at the 50% probability level. Hydrogen atoms are shown as spheres of arbitrary radius and C—H⋯N hydrogen bonds are indicated by dashed red lines.

Compound **1**·2CHCl_3_ crystallizes with two mol­ecules of tri­chloro­methane solvate per diazo-diphenanthroline (Fig. 1 ▸). The mol­ecule lies on a centre of symmetry and is essentially planar, the r.m.s. deviation from the plane of the atoms in the ring system is 0.259 Å. The mol­ecules lie parallel to the (5 7 15) or (5 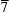 14) planes. There is a hydrogen bond between the alcohol and the diazo linker [O1⋯N2 = 2.540 (3) Å under symmetry operation −*x* + 2, −*y* + 1, −*z* + 1, Table 1 ▸] and the tri­chloro­methane mol­ecule is oriented by a bifurcated C—H⋯·N inter­action with the phenanthroline moiety [3.219 (3) and 3.136 (4) Å to N1 and N3, respectively]. The carbon atom of the tri­chloro­methane mol­ecule (C21) is 1.045 (3) Å from the mean plane of **1**·2CHCl_3_. There are 40 examples in the Cambridge Structural Database (CSD, Version 5.40, update of May 2019; Groom *et al.*, 2016 ▸) showing similar inter­actions between tri­chloro­methane and 1,10-phenanthroline derivatives and the geometry for **1**·2CHCl_3_ is typical of the group [mean Cl_3_CH⋯·N = 3.18 (5) Å].

The bond lengths indicate some delocalization through the central part of the mol­ecule. The C6—O1and C5—N2 bonds are short [1.318 (3) and 1.376 (3) Å, respectively] and the N=N bond, at 1.316 (4) Å, is significantly longer than in most free diazo mol­ecules [mean of 1.24 (4) Å for 2730 CSD entries].

## Supra­molecular features   

**Figure 2:**
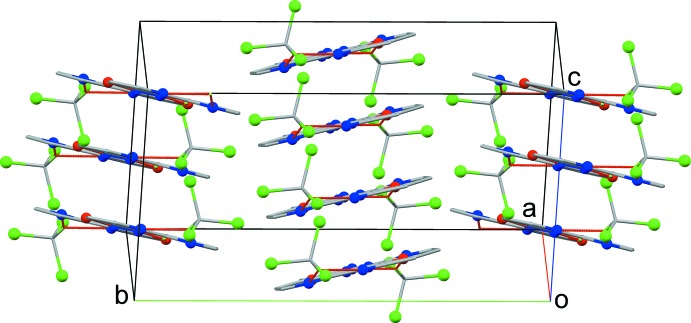
Unit-cell packing diagram viewed parallel to the plane of **1**·2CHCl_3_. Hydrogen atoms not involved in hydrogen bonding have been omitted for clarity.

**Figure 3:**
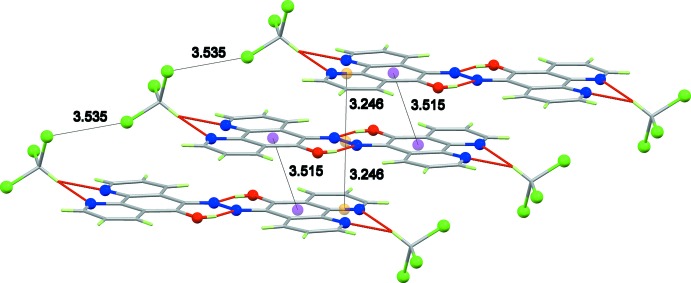
Principal inter­molecular inter­actions in **1**·2CHCl_3_. Purple spheres represent ring centroids and orange spheres show bond mid-points; distances in Å.

Fig. 2 ▸ shows the unit-cell packing, the mol­ecules lie parallel to the (5 7 15) or (5 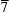 14) planes with a mean inter­planar distance of 3.228 (2) Å (under 1 − *x*, 1 − *y*, 1 − *z*) and the axis of the stack runs parallel to the *a* axis. The shortest ring centroid–centroid distance is 3.5154 (15) Å between the C_6_ rings; however, there is a more direct overlap between the diazo group and an imine group in the next layer (*ca *3.246 Å between the mid-point of the N=N bond and the mid-point of the C11=N3 bond under symmetry operation 1 − *x*, 1 − *y*, 1 − *z*) (Fig. 3 ▸). The most notable inter­actions between stacks are type 1 *R*—Cl⋯·Cl—*R* packing inter­actions (Mukherjee *et al.*, 2014 ▸; Cavallo *et al.*, 2016 ▸), the shortest Cl⋯Cl distance being 3.5353 (11) Å for Cl1⋯·Cl3 under symmetry operation −1 + *x*, *y*, *z*.

## Database survey   

There are three published examples of mol­ecules containing the 2,2′-di­hydroxy­azo­benzene core (Bougueria *et al.*, 2014 ▸; Evangelio *et al.*, 2008 ▸; Schilde *et al.*, 1994 ▸) and all of these are significantly less delocalized than **1**·2CHCl_3_.

## Spectroscopy studies of 1 in solution   

**Figure 4:**
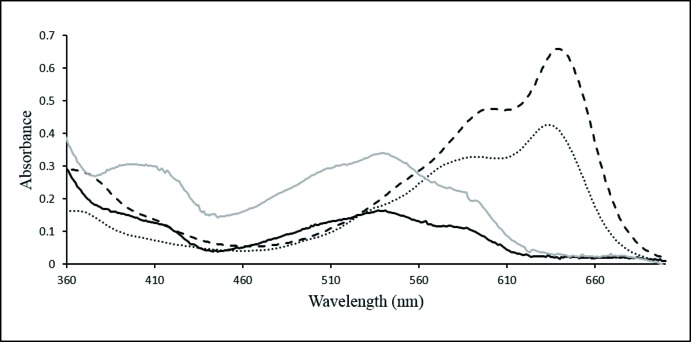
UV/vis spectra of **1**·2CHCl_3_ in (**^____^**) CHCl_3_, (- - - -) ethanol, (**^____^**) CH_2_Cl_2_, and (⋯⋯) methanol. The absorbance axis is ×10 for the CHCl_3_ and CH_2_Cl_2_ solutions.

Compound **1** has a very low solubility in all organic solvents investigated (CH_3_Cl, CH_2_Cl_2_, DMSO, CH_3_CN and alcohols). UV/vis spectra of **1**·2CHCl_3_ recorded in ethanol, methanol, CHCl_3_ and CH_2_Cl_2_ are given in Fig. 4 ▸. Compound **1** exhibits significant solvatochromism showing a broad band in CHCl_3_ and CH_2_Cl_2_ with λ_max_ at 543 nm. This band undergoes a bathochromic shift and separates into two bands in the alcohols with λ_max_ values at 643 nm and 600 nm in ethanol and at 636 nm and 591 nm in methanol. No accurate measurement of the extinction coefficient could be made as **1** was not fully soluble in the solvents and precipitation from the solvent occurred upon standing. The solubility of **1** was so low that only a very poorly resolved ^1^H NMR of the compound was obtained in *d* ^4^-methanol showing a series of peaks in the region expected for the phenanthroline H-atom signals and no definitive assignments of the peaks could be made. Inter­estingly, **1** was more soluble in strongly acidic solutions due to the protonation of one or more of the N atoms. Compound **1** dissolved in CF_3_COOD to form a bright-red solution. Six signals are observed in the ^1^H NMR spectrum in the region associated with the phenanthroline peaks. This finding is consistent with a compound which has a centre of symmetry, as found for the crystal structure, and suggests that at room temperature **1** remains in the *E* form in this solvent.

## Synthesis and crystallization   

**Figure 5:**
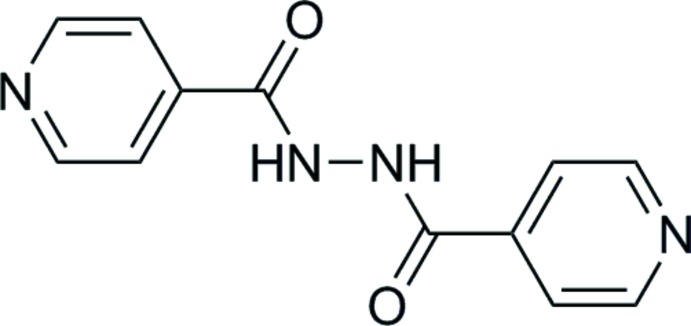
Chemical structure of **2**

The mechanism to form **1** from the reaction mixture is unclear; however, the formation of isonicotinic acid *N′*-(pyridine-4-carbon­yl)-hydrazide (**2**) from the mixture is an indication that radical chemistry is occurring. Isoniazid is well known to react to form radical species and these radicals are important in its role as an anti-tuberculosis drug (Timmins *et al.* 2006 ▸). Significantly one LC–MS study has shown that isoniazid will photo-degrade to form **2** (Fig. 5 ▸) and radical inter­mediates are proposed in its formation (Bhutani, 2007 ▸). Attempts were made to try to favour the formation of **1** using UV irradiation and the radical initiators azobisisobutyro­nitrile and 2,2′-azobis(2-amidino­propane) di­hydro­chloride but these were unsuccessful. However, although the reaction to form **1** was low yielding, attempts to make **1** by reaction of 6-amino-1,10-phenanthrolin-5-ol and 6-nitroso-1,10-phenananthrolin-5-ol using known conditions to form diazo compounds (Zhao *et al.* 2011 ▸) did not form the desired product. Studies are ongoing to improve the yield of the reaction to form **1**.

Phendione (0.210 g, 1.000 mmol) was added to solution of isoniazid (0.137 g, 1.000 mmol) in EtOH (25 cm^3^). *p*-Tolouene­sulfonic acid (10%, 0.02 g) was added and the solution refluxed for 6 h. The resulting suspension was filtered whilst hot and the filtrate allowed to stand in the dark overnight. Precipitated yellow (Z)-*N′*-(6-oxo-1,10-phenanthrolin-5(6*H*) yl­idene)isonicotinohydrazide (0.263 g, 0.799 mmol, 80%) was filtered off and the bright-orange filtrate was concentrated to *ca* 10 cm^3^ using rotary evaporation and then allowed to stand in the dark. Over a period of four weeks, the bright-orange filtrate changed to a dark-green suspension. This mixture was heated to reflux and filtered whilst hot to give a green filtrate (see below) and a dark-purple powder. The powder was dissolved in CHCl_3_ and allowed to crystallize over several days to produce dark-purple crystals of (*E*)-6,6′-(diazene-1,2-di­yl)bis­(1,10-phenanthrolin-5-ol) tri­chloro­meth­ane disolvate (**1**·2CHCl_3_) (0.026 g, 0.039 mmol, 6.2% based on isoniazid starting material). Upon leaving the above green filtrate to evaporate further white isonicotinic acid *N′*-(pyridine-4-carbon­yl)-hydrazide (compound **2**) precipitated. The supernatent was deca­nted off and the solid dissolved in hot acetone. This colourless solution was evaporated to dryness on a rotary evaporator to give **2** (0.015 g, 0.062 mmol, 12.4% based on isoniazid starting material).

Compound **1**: IR (ATR, cm^−1^) (1.2CHCl_3_): 3065, 2108, 1583, 1568, 1500, 1472, 1415, 1338, 1284, 1227, 1162, 1027, 795, 739, 717. ^1^H NMR (protonated-1) (CF_3_COOD, 500 MHz): δ 9.60 (*d*, *J* = 8.5 Hz, 2H), PhenH 9.56 (*d*, *J* = 8.5 Hz, 2H, PhenH), 9.47 (*d*, *J* = 5.0 Hz, 2H, PhenH), 9.37 (*d*, *J* = 5.0 Hz, 2H, PhenH), 8.57 (*dd*, *J* = 8.5, 5.0 Hz, 2H, PhenH), 8.38 (*dd*, *J* = 8.5, 5.0 Hz, 2H, PhenH), 4.12 (*s*, 2H, OH). Elemental analysis calculated for **1**·2CHCl_3_ (C_26_H_16_Cl_6_N_6_O_2_, 657.15 g mol^−1^): C 47.52, H 2.45, N 12.79%; found: C 47.73, H 2.52, N 12.77%.

Compound **2** has been previously reported (Quiroga *et al.*, 2008 ▸; Bhutani *et al.*, 2007 ▸) and the characterization data given are consistent with the data recorded in the present study.

Compound **2**: IR (KBr, cm^−1^) 3435, 3210, 3045, 1682, 1642, 1546, 1489, 1406, 1299, 838, 751. ^1^H NMR (CD_3_OD, 500 MHz): **δ** 8.80 (*d*, *J* = 5 Hz, 2H), 7.42 (*d*, *J* = 5 Hz, 2H). ^13^C NMR (CD_3_OD, 125 MHz): δ 165.6 (C=O), 150.0 (PyrC), 140.5 (PyrC), 121.8 (PyrC). MS: Calculated *m*/*z* for C_12_H_10_N_4_O_2_: (*M* + H)^+^ 243.0877; found: (*M* + H)^+^ 243.0882; difference (ppm): 2.15.

## Refinement   

Crystal data, data collection and structure refinement details are summarized in Table 2 ▸. The C-bound H atoms were included in calculated positions and treated as riding, with C—H = 0.95–1.00 Å and *U*_iso_(H) = 1.5*U*_eq_(C) for methyl H atoms or 1.2*U*_eq_(C) otherwise. The H atom (H1*A*) bonded to oxygen was located in a difference-Fourier map and its coord­inates were refined with *U*_iso_(H) = 1.5*U*_eq_(O).

## Supplementary Material

Crystal structure: contains datablock(s) I. DOI: 10.1107/S205698901900954X/jj2213sup1.cif

Structure factors: contains datablock(s) I. DOI: 10.1107/S205698901900954X/jj2213Isup2.hkl

Supporting information file. DOI: 10.1107/S205698901900954X/jj2213Isup3.cml

CCDC reference: 1937969

Additional supporting information:  crystallographic information; 3D view; checkCIF report

## Figures and Tables

**Table 1:** Hydrogen-bond geometry (Å, °)

*D*—H⋯*A*	*D*—H	H⋯*A*	*D*⋯*A*	*D*—H⋯*A*
O1—H1*A*⋯N2^i^	0.88 (3)	1.74 (3)	2.540 (3)	149 (3)
C21—H21⋯N1	1.00	2.36	3.219 (3)	143
C21—H21⋯N3	1.00	2.33	3.136 (4)	137

Symmetry code: (i) 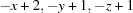.

**Table 2:** Experimental details

Crystal data	
Chemical formula	C_24_H_14_N_6_O_2_·2CHCl_3_
*M*_r_	657.15
Crystal system, space group	Monoclinic, *P*2_1_/*c*
Temperature (K)	150
*a*, *b*, *c* (Å)	5.9406 (7), 18.856 (2), 12.2375 (16)
β (°)	96.863 (4)
*V* (Å^3^)	1361.0 (3)
*Z*	2
Radiation type	Mo *K*α
μ (mm^−1^)	0.67
Crystal size (mm)	0.43 × 0.05 × 0.04

Data collection	
Diffractometer	Bruker–Nonius X8 APEXII CCD
Absorption correction	Multi-scan (*SADABS*; Sheldrick, 2012 ▸)
*T*_min_, *T*_max_	0.657, 0.745
No. of measured, independent and observed [*I* > 2σ(*I*)] reflections	18529, 2732, 2068
*R*_int_	0.045
(sin θ/λ)_max_ (Å^−1^)	0.620

Refinement	
*R*[*F* ^2^ > 2σ(*F* ^2^)], *wR*(*F* ^2^), *S*	0.044, 0.111, 1.03
No. of reflections	2732
No. of parameters	184
H-atom treatment	Heteroxyz
Δρ_max_, Δρ_min_ (e Å^−3^)	0.87, −0.59

Computer programs: *APEX2* and *SAINT* (Bruker, 2010 ▸), *SHELXT* (Sheldrick 2015*a* ▸), *SHELXL2018* (Sheldrick, 2015*b* ▸), *Mercury* (Macrae *et al.*, 2008 ▸) and *publCIF* (Westrip, 2010 ▸).

## supplementary crystallographic information

### Crystal data

**Table:**

C_24_H_14_N_6_O_2_·2CHCl_3_	*F*(000) = 664
*M**_r_* = 657.15	*D*_x_ = 1.604 Mg m^−^^3^
Monoclinic, *P*2_1_/*c*	Mo *K*α radiation, λ = 0.71073 Å
*a* = 5.9406 (7) Å	Cell parameters from 5155 reflections
*b* = 18.856 (2) Å	θ = 2.7–26.1°
*c* = 12.2375 (16) Å	µ = 0.67 mm^−^^1^
β = 96.863 (4)°	*T* = 150 K
*V* = 1361.0 (3) Å^3^	Lath, purple
*Z* = 2	0.43 × 0.05 × 0.04 mm

### Data collection

**Table:**

Bruker–Nonius X8 APEXII CCD diffractometer	2732 independent reflections
Radiation source: fine-focus sealed-tube	2068 reflections with *I* > 2σ(*I*)
Detector resolution: 9.1 pixels mm^-1^	*R*_int_ = 0.045
thin–slice ω and φ scans	θ_max_ = 26.2°, θ_min_ = 2.7°
Absorption correction: multi-scan (SADABS; Sheldrick, 2012)	*h* = −5→7
*T*_min_ = 0.657, *T*_max_ = 0.745	*k* = −23→23
18529 measured reflections	*l* = −15→14

### Refinement

**Table:**

Refinement on *F*^2^	Primary atom site location: dual
Least-squares matrix: full	Secondary atom site location: difference Fourier map
*R*[*F*^2^ > 2σ(*F*^2^)] = 0.044	Hydrogen site location: mixed
*wR*(*F*^2^) = 0.111	Heteroxyz
*S* = 1.02	*w* = 1/[σ^2^(*F*_o_^2^) + (0.0444*P*)^2^ + 1.8407*P*] where *P* = (*F*_o_^2^ + 2*F*_c_^2^)/3
2732 reflections	(Δ/σ)_max_ = 0.001
184 parameters	Δρ_max_ = 0.87 e Å^−^^3^
0 restraints	Δρ_min_ = −0.59 e Å^−^^3^

### Special details

**Table:**

Geometry. All esds (except the esd in the dihedral angle between two l.s. planes) are estimated using the full covariance matrix. The cell esds are taken into account individually in the estimation of esds in distances, angles and torsion angles; correlations between esds in cell parameters are only used when they are defined by crystal symmetry. An approximate (isotropic) treatment of cell esds is used for estimating esds involving l.s. planes.

### Fractional atomic coordinates and isotropic or equivalent isotropic displacement parameters (Å^2^)

**Table:**

	*x*	*y*	*z*	*U*_iso_*/*U*_eq_	
N1	0.3174 (4)	0.63628 (11)	0.66898 (18)	0.0216 (5)	
C1	0.3648 (5)	0.69741 (14)	0.6234 (2)	0.0240 (6)	
H1	0.269904	0.736919	0.632910	0.029*	
C2	0.5456 (5)	0.70706 (14)	0.5623 (2)	0.0255 (6)	
H2	0.572645	0.752054	0.531450	0.031*	
C3	0.6846 (4)	0.65044 (14)	0.5473 (2)	0.0219 (6)	
H3	0.810516	0.656051	0.507047	0.026*	
C4	0.6383 (4)	0.58421 (13)	0.5923 (2)	0.0174 (5)	
C5	0.7702 (4)	0.52078 (13)	0.5757 (2)	0.0177 (5)	
N2	0.9396 (3)	0.52817 (11)	0.50912 (17)	0.0195 (5)	
C6	0.7175 (4)	0.45623 (14)	0.6231 (2)	0.0195 (5)	
O1	0.8303 (3)	0.39726 (10)	0.61010 (16)	0.0246 (4)	
H1A	0.937 (6)	0.4088 (17)	0.569 (3)	0.037*	
C7	0.5313 (4)	0.45226 (13)	0.6889 (2)	0.0191 (5)	
C8	0.4726 (5)	0.38783 (14)	0.7366 (2)	0.0239 (6)	
H8	0.554738	0.345644	0.726050	0.029*	
C9	0.2948 (5)	0.38714 (15)	0.7985 (2)	0.0274 (6)	
H9	0.251833	0.344474	0.831710	0.033*	
C10	0.1781 (5)	0.45015 (15)	0.8118 (2)	0.0253 (6)	
H10	0.057433	0.449191	0.856178	0.030*	
N3	0.2257 (4)	0.51143 (12)	0.76615 (18)	0.0224 (5)	
C11	0.4006 (4)	0.51294 (13)	0.7050 (2)	0.0182 (5)	
C12	0.4522 (4)	0.58002 (13)	0.6541 (2)	0.0185 (5)	
C21	0.0382 (5)	0.64497 (15)	0.8784 (2)	0.0271 (6)	
H21	0.087592	0.621547	0.811921	0.032*	
Cl1	−0.23375 (13)	0.61485 (6)	0.89683 (7)	0.0493 (3)	
Cl2	0.03861 (18)	0.73803 (4)	0.85952 (7)	0.0508 (3)	
Cl3	0.23081 (12)	0.62261 (4)	0.99506 (6)	0.0333 (2)	

### Atomic displacement parameters (Å^2^)

**Table:**

	*U*^11^	*U*^22^	*U*^33^	*U*^12^	*U*^13^	*U*^23^
N1	0.0234 (11)	0.0211 (12)	0.0208 (12)	0.0047 (9)	0.0050 (9)	−0.0007 (9)
C1	0.0290 (14)	0.0208 (15)	0.0229 (14)	0.0070 (11)	0.0065 (11)	−0.0003 (11)
C2	0.0330 (15)	0.0159 (13)	0.0279 (15)	0.0000 (11)	0.0054 (12)	0.0012 (11)
C3	0.0231 (13)	0.0216 (14)	0.0219 (14)	−0.0011 (11)	0.0064 (11)	−0.0016 (11)
C4	0.0192 (13)	0.0169 (13)	0.0160 (12)	0.0001 (10)	0.0008 (10)	−0.0016 (10)
C5	0.0158 (12)	0.0196 (13)	0.0169 (13)	0.0011 (10)	−0.0011 (10)	−0.0021 (10)
N2	0.0180 (11)	0.0213 (11)	0.0193 (11)	0.0023 (8)	0.0020 (9)	−0.0036 (9)
C6	0.0192 (13)	0.0203 (14)	0.0180 (13)	0.0023 (10)	−0.0011 (10)	−0.0020 (10)
O1	0.0258 (10)	0.0197 (10)	0.0293 (11)	0.0053 (8)	0.0072 (8)	0.0007 (8)
C7	0.0212 (13)	0.0203 (13)	0.0151 (13)	0.0003 (10)	−0.0007 (10)	0.0007 (10)
C8	0.0277 (14)	0.0199 (14)	0.0236 (14)	0.0023 (11)	0.0004 (11)	0.0027 (11)
C9	0.0319 (15)	0.0274 (15)	0.0230 (14)	−0.0046 (12)	0.0036 (12)	0.0063 (12)
C10	0.0244 (14)	0.0290 (15)	0.0236 (14)	−0.0027 (11)	0.0077 (11)	0.0032 (12)
N3	0.0235 (11)	0.0250 (12)	0.0190 (11)	−0.0009 (9)	0.0039 (9)	0.0005 (9)
C11	0.0207 (13)	0.0198 (13)	0.0141 (12)	0.0010 (10)	0.0016 (10)	−0.0020 (10)
C12	0.0199 (13)	0.0196 (13)	0.0156 (13)	0.0008 (10)	0.0008 (10)	−0.0015 (10)
C21	0.0290 (15)	0.0290 (16)	0.0242 (15)	0.0014 (12)	0.0077 (12)	−0.0021 (12)
Cl1	0.0258 (4)	0.0875 (7)	0.0346 (5)	−0.0121 (4)	0.0037 (3)	−0.0095 (4)
Cl2	0.0818 (7)	0.0295 (4)	0.0438 (5)	0.0156 (4)	0.0196 (5)	0.0009 (4)
Cl3	0.0273 (4)	0.0379 (4)	0.0339 (4)	−0.0051 (3)	0.0008 (3)	0.0002 (3)

### Geometric parameters (Å, º)

**Table:**

N1—C1	1.326 (3)	O1—H1A	0.88 (3)
N1—C12	1.355 (3)	C7—C11	1.409 (4)
C1—C2	1.392 (4)	C7—C8	1.409 (4)
C1—H1	0.9500	C8—C9	1.371 (4)
C2—C3	1.375 (4)	C8—H8	0.9500
C2—H2	0.9500	C9—C10	1.395 (4)
C3—C4	1.405 (4)	C9—H9	0.9500
C3—H3	0.9500	C10—N3	1.328 (3)
C4—C12	1.414 (3)	C10—H10	0.9500
C4—C5	1.457 (3)	N3—C11	1.352 (3)
C5—N2	1.376 (3)	C11—C12	1.459 (4)
C5—C6	1.400 (4)	C21—Cl1	1.752 (3)
N2—N2^i^	1.316 (4)	C21—Cl2	1.770 (3)
C6—O1	1.318 (3)	C21—Cl3	1.771 (3)
C6—C7	1.446 (4)	C21—H21	1.0000
			
C1—N1—C12	117.7 (2)	C8—C7—C6	121.2 (2)
N1—C1—C2	123.8 (2)	C9—C8—C7	118.8 (2)
N1—C1—H1	118.1	C9—C8—H8	120.6
C2—C1—H1	118.1	C7—C8—H8	120.6
C3—C2—C1	118.9 (3)	C8—C9—C10	118.9 (3)
C3—C2—H2	120.5	C8—C9—H9	120.6
C1—C2—H2	120.5	C10—C9—H9	120.6
C2—C3—C4	119.3 (2)	N3—C10—C9	123.9 (2)
C2—C3—H3	120.3	N3—C10—H10	118.0
C4—C3—H3	120.3	C9—C10—H10	118.0
C3—C4—C12	117.4 (2)	C10—N3—C11	117.9 (2)
C3—C4—C5	122.8 (2)	N3—C11—C7	122.2 (2)
C12—C4—C5	119.8 (2)	N3—C11—C12	118.0 (2)
N2—C5—C6	123.3 (2)	C7—C11—C12	119.8 (2)
N2—C5—C4	116.3 (2)	N1—C12—C4	122.7 (2)
C6—C5—C4	120.4 (2)	N1—C12—C11	117.6 (2)
N2^i^—N2—C5	118.1 (3)	C4—C12—C11	119.6 (2)
O1—C6—C5	122.8 (2)	Cl1—C21—Cl2	110.73 (16)
O1—C6—C7	117.2 (2)	Cl1—C21—Cl3	109.64 (15)
C5—C6—C7	120.0 (2)	Cl2—C21—Cl3	109.29 (16)
C6—O1—H1A	106 (2)	Cl1—C21—H21	109.1
C11—C7—C8	118.3 (2)	Cl2—C21—H21	109.1
C11—C7—C6	120.4 (2)	Cl3—C21—H21	109.1

Symmetry code: (i) −*x*+2, −*y*+1, −*z*+1.

### Hydrogen-bond geometry (Å, º)

**Table:**

*D*—H···*A*	*D*—H	H···*A*	*D*···*A*	*D*—H···*A*
O1—H1*A*···N2^i^	0.88 (3)	1.74 (3)	2.540 (3)	149 (3)
C21—H21···N1	1.00	2.36	3.219 (3)	143
C21—H21···N3	1.00	2.33	3.136 (4)	137

Symmetry code: (i) −*x*+2, −*y*+1, −*z*+1.
